# Supplementary material for: JND4135, a New Type II TRK Inhibitor, Overcomes TRK xDFG and Other Mutation Resistance In Vitro and In Vivo
Source: Molecules. 2022 Oct 1;27(19):6500. doi: 10.3390/molecules27196500 (PMC9570838; doi:10.3390/molecules27196500)
Supplement: Supplementary file 1 [file molecules-27-06500-s001.zip › Figures S1-S4.pdf]

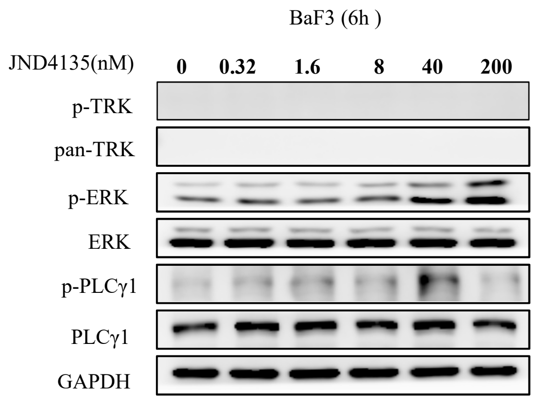

Figure Sl. Effects of JND4135 on TRK signal pathway in BaF3 cells（6h)
Cells were treated with 0-200 nmol/L of JND4135 for 6 h, then harvested and lysed. Cell lysates were separated by sodium dodecyl sulfate-polyacrylamide gel electrophoresis (SDS-PAGE) and analyzed by Western blot .

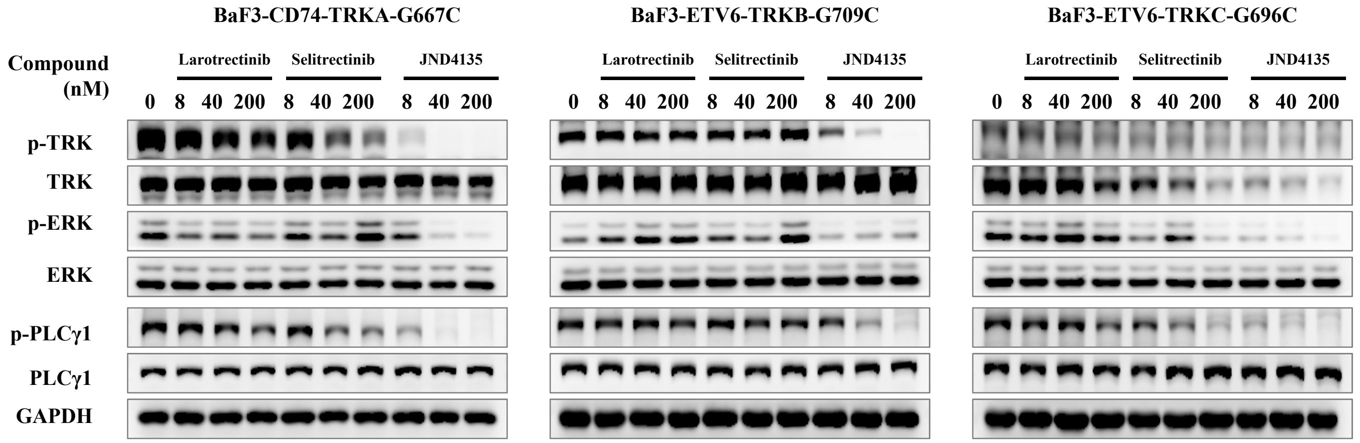

Figure S2. JND4135 effectively suppresses xDFG mutant TRK signal pathway in stable BaF3 models（6h)
Cells were treated with 8, 40 and 200 nmol/L of JND4135 for 6 h, then harvested and lysed. Cell lysates were separated by sodium dodecyl sulfate-polyacrylamide gel electrophoresis (SDS-PAGE) and analysed by Western blot for (p)Trk, (p)PLC-γ1and (p) Erk. GAPDH was used as a loading control.

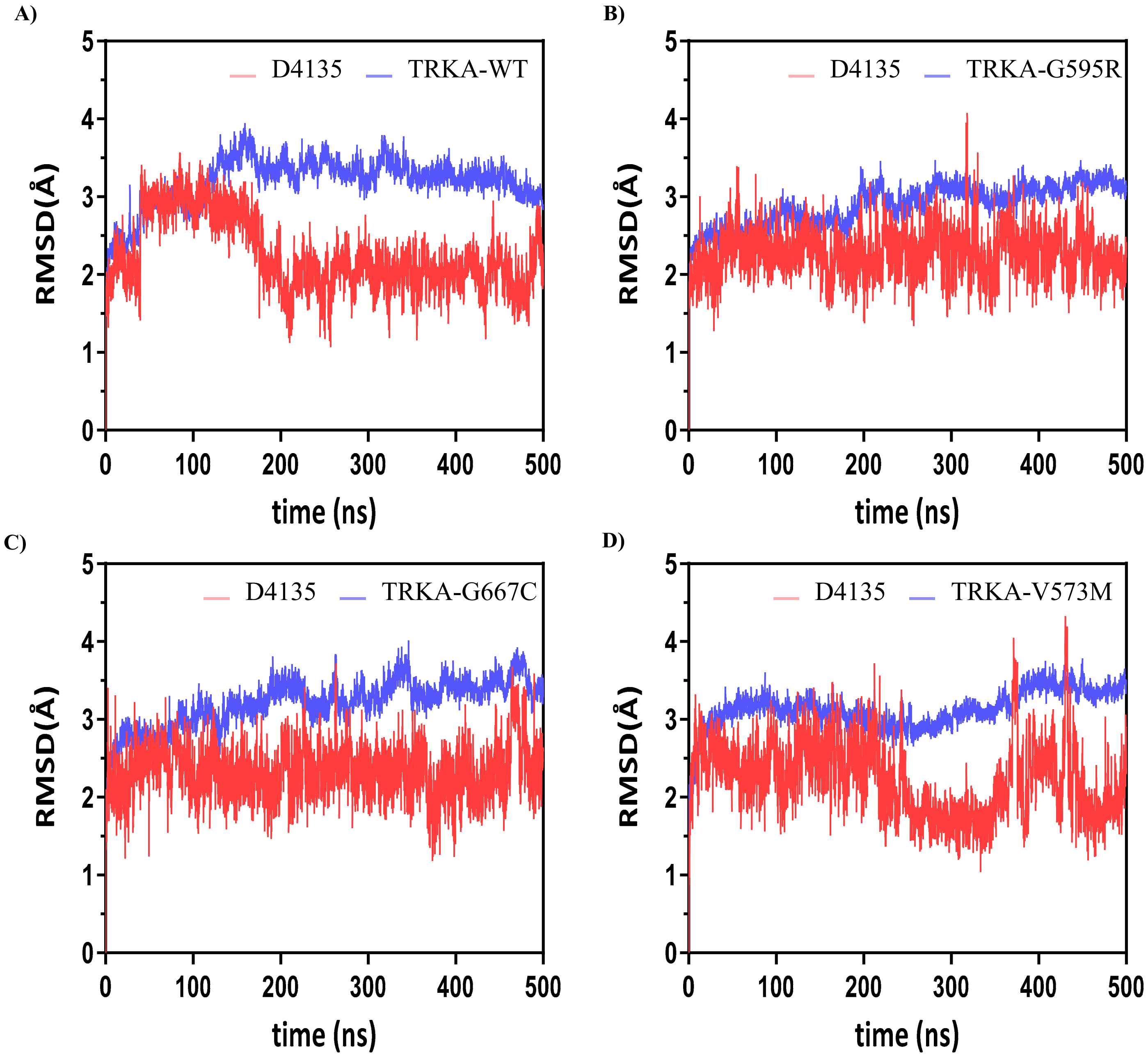

Figure S3. RMSD value of JND4135 with TRKA (A) ,TRKA-G595R (B), TRKA-G667C (C) and TRKA-V573M (D) proteins during the 500 ns MD simulations.

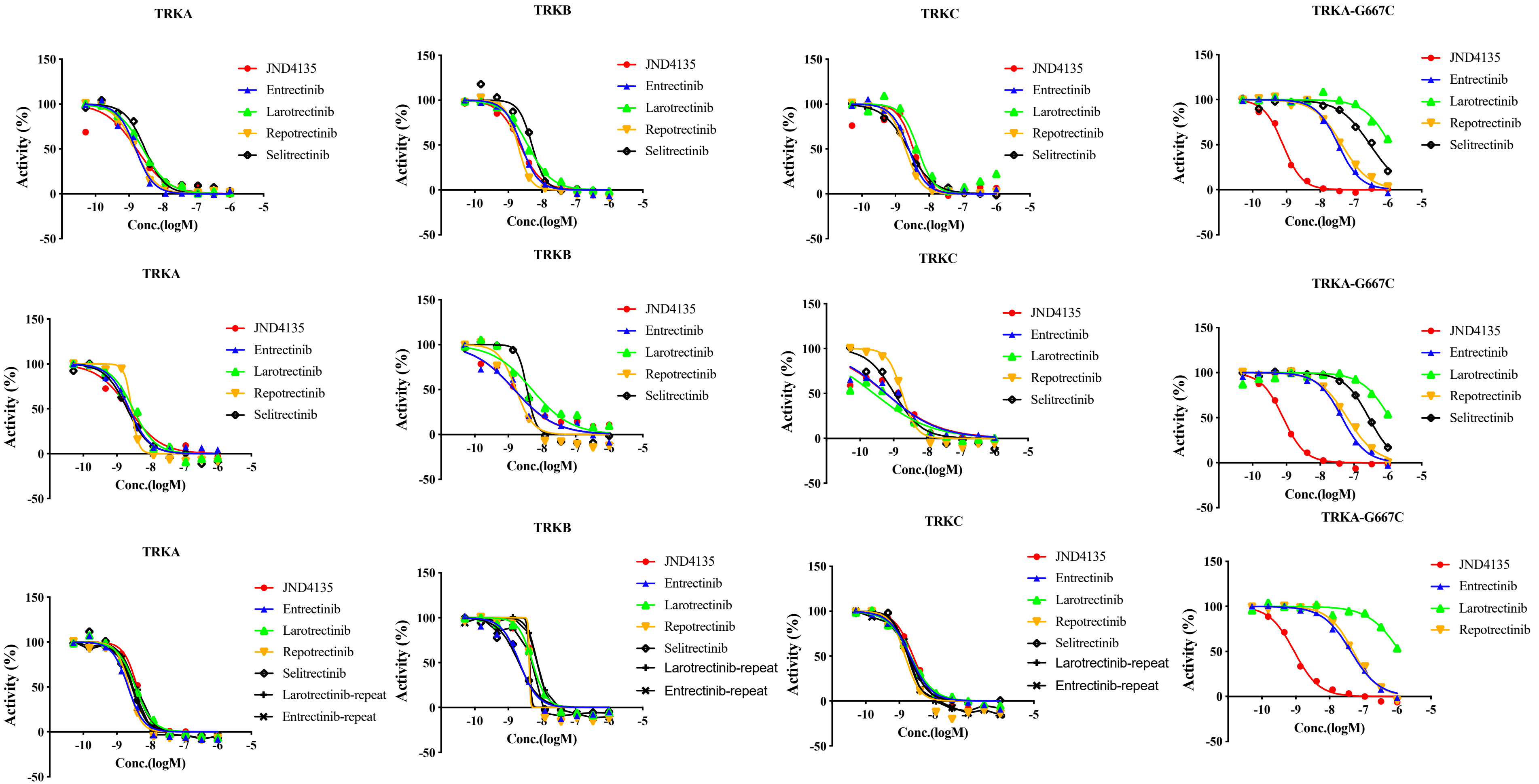

Figure S4. Kinase inhibitory activities of JND4135 against TRKA, TRKB, TRKC and TRKA-G667C. Trks activity experiments were performed using the FRET-based Z0-Lyte assay according to the manufacturer's instructions.
