# Supplementary material for: JND4135, a New Type II TRK Inhibitor, Overcomes TRK xDFG and Other Mutation Resistance In Vitro and In Vivo
Source: Molecules. 2022 Oct 1;27(19):6500. doi: 10.3390/molecules27196500 (PMC9570838; doi:10.3390/molecules27196500)
Supplement: Supplementary file 1 [file molecules-27-06500-s001.zip › Tables S1-S4.pdf]

Table S1. In vitro inhibitory activities of JND4135 against TrkA, TrkB and TrkC kinases.

Kinase IC_{50} (nM)	TRKA	TRKB	TRKC	TRKA(G667C)
JND4135	1.75	2.23	3.42	0.79
JND4135	2.54	2.12	3.03	0.80
JND4135	4.06	5.23	2.57	0.90
**AV±SD**	**2.79±1.17**	**3.19±1.76**	**3.01±0.43**	**0.83±0.06**
Entrectinib	1.56	2.44	2.48	34.31
Entrectinib	2.32	1.94	2.71	44.04
Entrectinib	2.14	2.17	2.09	42.69
Entrectinib	2.97	5.03	1.77	/
**AV±SD**	**2.25±0.58**	**2.89±1.44**	**2.26±0.41**	**40.35±5.27**
Larotrectinib	2.57	3.80	4.63	1225.00
Larotrectinib	3.00	3.96	2.44	1149.00
Larotrectinib	3.54	5.27	2.16	1214.00
Larotrectinib	3.66	7.84	2.04	/
**AV±SD**	**3.20±0.50**	**5.22±1.87**	**2.82±1.22**	**1196±41.07**
Repotrectinib	1.51	1.92	1.99	43.69
Repotrectinib	1.75	1.69	1.77	62.60
Repotrectinib	2.30	4.94	1.48	50.35
**AV±SD**	**1.85±0.41**	**2.85±1.82**	**1.75±0.25**	**52.21±9.59**
Selitrectinib	2.78	2.06	2.00	300.00
Selitrectinib	2.98	5.06	2.41	274.60
Selitrectinib	2.02	3.62	1.20	/
**AV±SD**	**2.59±0.44**	**3.58±1.40**	**1.87±0.54**	**287.3±17.96**

Trks activity experiments were performed using the FRET-based Z0-Lyte assay according to the manufacturer's instructions. The data are mean values from at least two independent experiments. Concentration-inhibitory curves were shown in Figure S4.

Table S2. Affinity activity parameters of JND 135 and reported TRKs inhibitors using BLI assays.

Compound	TRKC			$TRKC^{G696C}$		
	K_d (nM)	K_{on} (1/Ms)	K_{off} (1/s)	K_d (nM)	K_{on} (1/Ms)	K_{off} (1/s)
JND4135	2.57	1.09E+05	2.78E-04	3.68	3.67E+05	1.35E-03
Larotrectinib	5.38	4.18E+05	2.25E-03	37.8	9.94E+05	3.76E-02
Entrectinib	3.47	6.30E+05	2.18E-03	26.2	3.73E+05	9.77E-03
Selitrectinib	1.74	6.09E+05	1.06E-03	14.9	8.89E+05	1.33E-02
Repotrectinib	1.04	1.47E+06	1.53E-03	35	7.29E+05	1.95E-02

Affinity activity asssay were performed using biolayer interferometry according to the manufacturer's instructions. The data are mean values from at least three independent experiments

\

Table S3. **Muti-target kinase inhibitors overcomes TRK mutant resistance in BaF3 cell proliferation assays**

IC50(nM±SD)		Nintedanib	Sitravatinib	Altiratinib	Cabozantinib	Ponatinib	Foretinib	Merestinib
Parental	BaF3(+IL3)	3920±358	1460±332	6756±1368	7666±1277	1100±244	490±101	5519±2948
WT	MPRIP-TRKA	82.0±31.1	14.5±3.0	9.3±1.4	107±30.6	50.1±2.2	5.5±0.4	27.4±4.0
	CD74-TRKA	106±22.2	25.7±3.6	14.7±1.0	160±12.8	72.6±13.2	13.3±2.4	51.8±9.6
	QKI-TRKB	514±251	30.1±12.0	5.1±4.8	89.9±26.8	26.9±11.0	16±8.7	21.5±9.3
	ETV6-TRKB	918±260	142±8.2	38.0±7.1	577±127	113±13.5	126±27.5	181±28.8
	EML4-TRKC	1336±578	85.1±36.6	31.8±14.5	314±36.7	128±30.0	61.8±32.7	212±43.6
	ETV6-TRKC	672±16.3	28.7±10.1	13.6±3.7	152±8.3	61.2±17.7	24.2±11.5	485±202
Solvent Front	CD74-TRKA-G595R	857±319	676±85.8	1572±120	2106±389	324±43.8	281±44.1	1071±207
	ETV6-TRKB-G639R	1152±478	394±197	406±258	983±326	99.0±48.8	285±123	1002±504
	ETV6-TRKC-G623R	746±288	201±65.7	109±57.4	475±186	241±30.7	250±93.5	1165±781
ATP site	CD74-TRKA-V573M	710±266	7.5±2.8	6.1±2.3	36.3±1.6	56.8±30.5	6.3±5.1	6.5±3.5
	ETV6-TRKB-V617M	2318±779	13.7±0.5	5.6±1.7	41.6±4	38.5±4.6	9.3±2.9	3.8±0.6
	ETV6-TRKC-V601M	2455±221	6.9±2.2	4.1±2.7	22.1±9.7	26.1±11.6	5.7±2.6	12.6±4.9
Gatekeeper	CD74-TRKA-F589L	15.8±3.1	8.3±1.8	7.5±2.1	62.8±12.4	50.5±13.9	6.6±1.4	19.6±13.3
	ETV6-TRKB-F633L	496±328	169±17.2	67.0±13.2	361±160	157±42.2	126±28.5	377±198
	ETV6-TRKC-F617L	166±74.7	5.7±1.4	3.7±1.3	34.4±4.7	5.7±1.9	5.5±5.1	475±410
xDFG	CD74-TRKA-G667C	5.1±0.8	0.9±0.1	2.3±0.3	4.5±1.2	1.9±0.9	1.0±0.2	1.3±0.2
	ETV6-TRKB-G709C	175±24.7	22.1±4.2	22.5±0.9	34.8±4.0	13.6±3.5	21.4±8.0	19.0±7.0
	ETV6-TRKC-G696C	30.5±12.5	1.1±1.0	1.0±0.7	2.1±1.6	4.6±3.4	1.4±1.2	2.5±1.8

The anti-proliferative activities of the compounds were evaluated using CCK-8 assay. The data were means from at least three independent experiments.

Table S4. Pharmacokinetic parameters of JND4135 in rats.

Number	$t_{1/2}$	T_{max}	C_{max}	$AUC_{(0-t)}$	$AUC_{(0-\infty)}$	$MRT_{(0-t)}$	$MRT_{(0-\infty)}$	Vz	CL	F
	h	h	ng/mL	h*ng/mL	h*ng/mL	h	h	mL/kg	mL/h/kg	%
JND4135-IV-5 mg/kg										
301	3.98	0.083	1538.70	642.55	653.90	0.48	0.71	43906.78	7646.47	-
302	1.06	0.083	2048.00	800.54	803.31	0.43	0.46	9527.23	6224.27	-
303	1.04	0.083	2318.40	970.85	974.15	0.46	0.49	7679.30	5132.68	-
Mean	**2.03**	**0.083**	**1968.37**	**804.65**	**810.45**	**0.46**	**0.55**	**20371.11**	**6334.47**	**-**
SD	**1.69**	**0.000**	**395.90**	**164.19**	**160.25**	**0.03**	**0.14**	**20403.43**	**1260.51**	**-**
JND4135-PO-25 mg/kg										
401	4.23	2.00	73.11	428.62	438.53	5.46	6.02	-	-	10.65
402	5.78	2.00	51.90	390.48	409.20	6.16	7.35	-	-	9.71
403	4.31	2.00	64.89	475.38	487.81	5.99	6.61	-	-	11.82
Mean	**4.77**	**2.0**	**63.30**	**431.49**	**445.18**	**5.87**	**6.66**	-	-	**10.72**
SD	**0.87**	**0.0**	**10.69**	**42.52**	**39.72**	**0.36**	**0.67**	-	-	**1.06**
